# CellRomeR: an R package for clustering cell migration phenotypes from microscopy data

**DOI:** 10.1093/bioadv/vbaf069

**Published:** 2025-04-04

**Authors:** Iivari Kleino, Mats Perk, António G G Sousa, Markus Linden, Julia Mathlin, Daniel Giesel, Paulina Frolovaite, Sami Pietilä, Sini Junttila, Tomi Suomi, Laura L Elo

**Affiliations:** Turku Bioscience Centre, University of Turku and Åbo Akademi University, Turku, FI-20520, Finland; Turku Bioscience Centre, University of Turku and Åbo Akademi University, Turku, FI-20520, Finland; Turku Bioscience Centre, University of Turku and Åbo Akademi University, Turku, FI-20520, Finland; Turku Bioscience Centre, University of Turku and Åbo Akademi University, Turku, FI-20520, Finland; Turku Bioscience Centre, University of Turku and Åbo Akademi University, Turku, FI-20520, Finland; Institute of Biomedicine, University of Turku, Turku, FI-20014, Finland; Turku Bioscience Centre, University of Turku and Åbo Akademi University, Turku, FI-20520, Finland; Turku Bioscience Centre, University of Turku and Åbo Akademi University, Turku, FI-20520, Finland; Turku Bioscience Centre, University of Turku and Åbo Akademi University, Turku, FI-20520, Finland; Turku Bioscience Centre, University of Turku and Åbo Akademi University, Turku, FI-20520, Finland; Turku Bioscience Centre, University of Turku and Åbo Akademi University, Turku, FI-20520, Finland; Turku Bioscience Centre, University of Turku and Åbo Akademi University, Turku, FI-20520, Finland; Institute of Biomedicine, University of Turku, Turku, FI-20014, Finland

## Abstract

**Motivation:**

The analysis of cell migration using time-lapse microscopy typically focuses on track characteristics for classification and statistical evaluation of migration behaviour. However, considerable heterogeneity can be seen in cell morphology and microscope signal intensity features within the migrating cell populations.

**Results:**

To utilize this information in cell migration analysis, we introduce here an R package CellRomeR, designed for the phenotypic clustering of cells based on their morphological and motility features from microscopy images. Utilizing machine learning techniques and building on an iterative clustering projection method, CellRomeR offers a new approach to identify heterogeneity in cell populations. The clustering of cells along the migration tracks allows association of distinct cellular phenotypes with different cell migration types and detection of migration patterns associated with stable and unstable cell phenotypes. The user-friendly interface of CellRomeR and multiple visualization options facilitate an in-depth understanding of cellular behaviour, addressing previous challenges in clustering cell trajectories using microscope cell tracking data.

**Availability and implementation:**

CellRomeR is available as an R package from https://github.com/elolab/CellRomeR.

## 1 Introduction

Cell migration types and patterns are associated with various biological processes in living organisms (SenGupta *et al.* 2021, Alonso-Matilla *et al.* 2025). Considerable heterogeneity can be found even within a single-cell type with cells exhibiting diverse phenotypic states, including different migratory, proliferative, and quiescent states. These manifest as morphological and motility characteristics recorded with time-lapse microscopy setups. Recent breakthroughs in automated cell imaging technologies have increased the size and complexity of imaging datasets, spurring progress in tools for segmentation and tracking of cells from a series of live cell microscopy images, such as TrackMate (Tinevez *et al.* 2017) and CellTraxx (Holme *et al.* 2023). These cell segmentation tools extract morphological and migratory variables from the raw data enabling the downstream analysis of cells’ migratory types commonly studied at migration trajectory variation level (Masuzzo *et al.* 2016, Hu *et al.* 2023). These developments have greatly improved our understanding of cell migration and cell behaviour and have opened new avenues for detailed exploration of these migratory characteristics (Emami *et al.* 2021).

Study of biological variation at single-cell level is a recent development in many biological modalities (Kleino *et al.* 2022, Heumos *et al.* 2023). The microscopy imaging data per cell consisting of light signal intensity values and segmentation derived morphology features for large numbers of cells could allow study of cell heterogeneity in cell imaging settings. Clustering cells based on these variables could enable classification of the cells into distinct phenotypic groups and association of morphological or other phenotypic features to cell migration types and patterns. Furthermore, the classifications could be leveraged to investigate the stability and dynamic transitions of cell phenotypes during microscopy assays. For example, clustering can help determine whether cells maintain stable phenotypes over time or undergo systematic changes in phenotype during the migration. Additionally, clustering can reveal phenotypic alterations due to cell cycle progression or highlight potential cell segmentation and track-linking errors.

Typical approaches still often involve averaging the tracking and morphological features at the population level, which can mask the heterogeneity and contributions of different cell subsets. In recognition of this issue, emerging tools aim to identify subsets of similarly behaving cells from complex microscopy data, providing a more detailed understanding of cellular behaviour. The advancement in cell population analysis has been greatly facilitated by tools such as Traject3D (Freckmann *et al.* 2022), CellPhe (Wiggins *et al.* 2023), and cellPLATO (Shannon *et al.* 2024). However, despite these advancements, challenges persist in their usability, visualization capabilities, and reliance on basic clustering techniques, which may not be optimally suited for every dataset. This highlights the ongoing need for integrating cell migration data analysis with advanced cell population clustering approaches.

Here, we introduce CellRomeR, an R package designed to meet these needs. CellRomeR presents a new approach for phenotypic clustering of cells based on their migration, morphology, and behaviour at each time point. It leverages post-segmentation and tracking data from TrackMate and is built on our iterative clustering projection (ICP) method, originally developed for the identification of cell populations in single-cell transcriptomics data (Smolander *et al.* 2021). This self-supervised machine learning tool employs logistic regression and clustering similarity comparisons to iteratively cluster data, facilitating the discovery of subtle differences through probabilistic feature extraction. Although there are notable differences between single-cell transcriptomics and live cell imaging data, both fields can benefit from such a data-driven iterative method.

**Figure 1.**
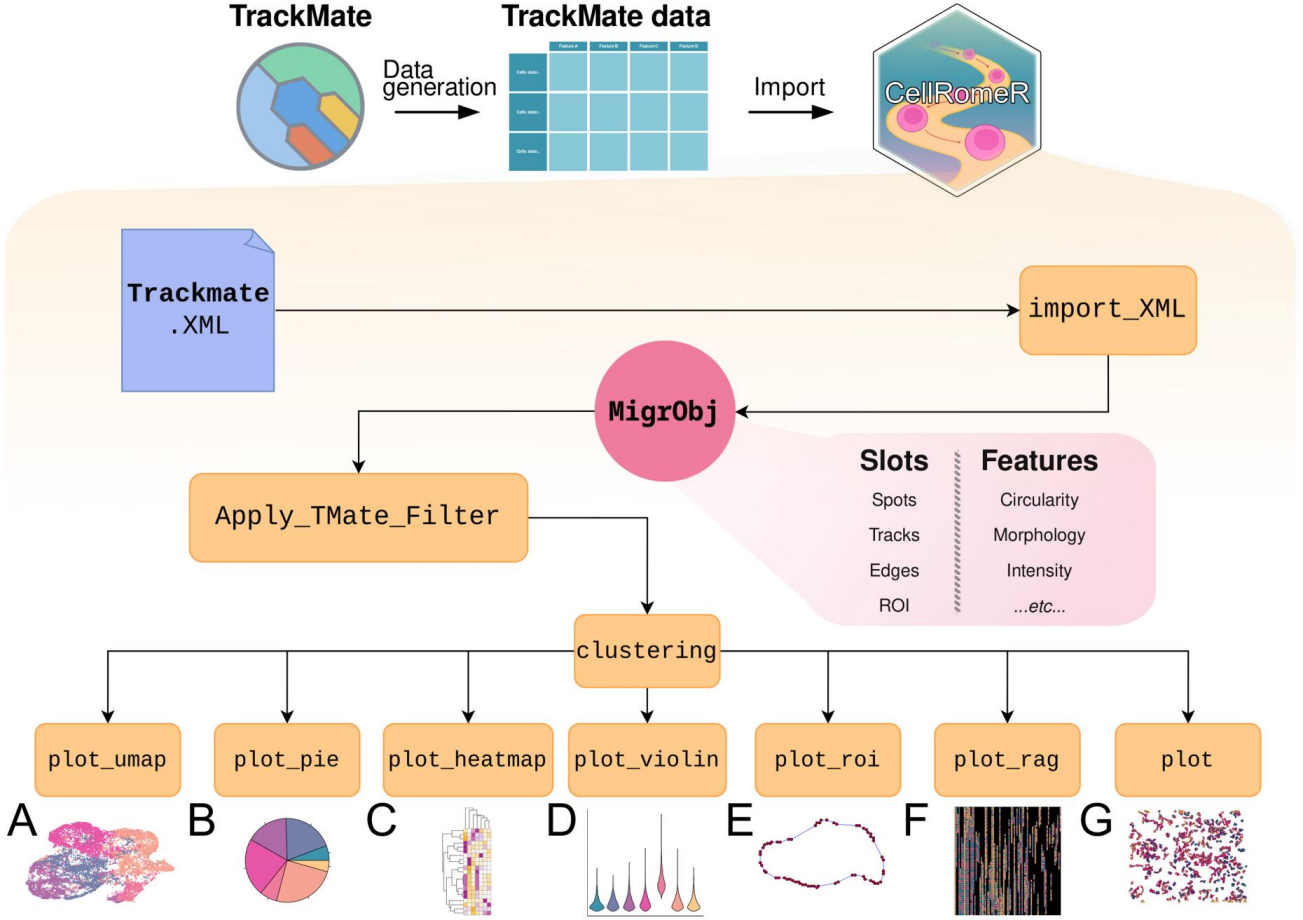
Schematic illustration of the CellRomeR workflow. (A–G) Output examples of CellRomeR's visualisation functions.

Moreover, CellRomeR offers several advantages, including diverse options for visualizing both the data and the results. The package is user-friendly, featuring intuitive functions and a new R migration data object to hold cell migration data and the analysis results. It also provides a step-by-step vignette, together with practical examples, to facilitate data analysis. A schematic illustration of the CellRomeR workflow is shown in Fig. 1.

## 2 Methods and implementation

### 2.1 Input data and migration data object

CellRomeR takes as input live cell migration tracking data that has been produced from microscopy image sets using cell segmentation and linking cells in sequential images to cell tracks. The current CellRomeR implementation is compatible with morphological and motility data exported from the TrackMate tool as XML files (Tinevez *et al.* 2017). The workflow in TrackMate starts with cell segmentation which generates region-of-interest (ROI) masks that define the boundaries of individual cells. These ROI masks are then used to derive series of *xy*-coordinates (roi_points) that outline the segmented regions. Based on these coordinates, numerical morphological values are computed to describe the shape characteristics of each detected cell or spot within the source image series. Additionally, the ROI-mask areas are leveraged alongside the signal intensities from multiple light channels in the microscope images to calculate a comprehensive set of light intensity statistics for each detected cell. Cell movement tracks in TrackMate are constructed using algorithms that utilize spatial coordinates to link cells across sequential images, first forming edges and subsequently assembling complete tracks. This process generates edge and track matrices, which contain computed characteristics such as speed and movement directionality. The resulting morphological and technical variables form a structured numerical data matrix that characterizes every detected cell, allowing downstream analyses in CellRomeR.

The function *import_XML(file)* reads in the data and converts it into the MigrDat cell migration data object, which enables data and result storing in a structured format. The MigrDat object includes features of the complete migration pathway, containing separate slots for spots, edges, and tracks, stored in a data.table format. The slot for spots contains details about the individual cell snapshots at each time point; the slot for edges links the adjacent spots; and the slot for tracks contains the cell trajectory features over time. The spots data.table in MigrObject can be supplemented with any data vectors matching the spot IDs and used alongside the original TrackMate data for cell clustering.

The CellRomeR analysis results are stored to their dedicated slots in MigrDat object, which can be saved and shared in a compressed format, such as R data. The cell/spot filtering done in TrackMate is also stored in MigrDat object and it is used with CellRomeR *Apply_TMate_Filter(MigrDat)* function, which removes TrackMate filtered cells from the analysis. This is useful as the data imported from native TrackMate XML files still contain information of all the cells, including poor-quality and non-linked cells.

### 2.2 Iterative clustering projection

The cell migration data consist of directly recorded technical features and derived morphological features in numerical format for each cell. These features are used in CellRomeR to group the cells into different phenotypic clusters. CellRomeR lets the user flexibly choose the features for cell clustering. The clustering is done using the function *clustering(MigrObj)*, which is built on our recently introduced ILoReg tool, initially developed for identifying fine-grained cell populations in single-cell transcriptomics data (Smolander *et al.* 2021). The method was chosen as we expect that the ability to distinguish fine-grained clusters will be equally important for analysing single-cell migration data.

At its core, ILoReg runs the Iterative Clustering Projection (ICP) algorithm, a self-learning ensemble method that leverages L1-regularized logistic regression to identify key features that maximize the discrimination of cell populations. Briefly, the process begins by randomly assigning a cluster to each cell. In the second step, 30% of the cells per cluster are selected to train a logistic regression model with L1-regularization. The third step uses this model to predict the cluster identities for all cells. In the fourth step, the agreement between the predicted clustering (*S*′) and the modelled clustering (*S*) is assessed using the adjusted rand index (ARI). If the ARI increases (starting from 0), *S*′ is used for the next iteration, replacing *S*; otherwise, *S* is retained. Steps 2–4 are repeated until ICP converges to a stable clustering result, where ARI approaches 1. The final output consists of a set of probability matrices, each with *N* cells × *K* clusters, which are concatenated to compute a consensus PCA. Finally, ILoReg performs hierarchical clustering on the computed PCA using Ward’s method and computes Uniform Manifold Approximation and Projection (UMAP) coordinates for visual inspection in a reduced-dimensional space. For accurate sample comparison, a feature-wise data normalization is applied during the clustering process.

### 2.3 Visualization functions

CellRomeR offers various data and result visualization options. The clustering can be visualized in a low-dimensional space with the function *plot_umap(MigrObj)*, which projects the features of cells using the UMAP coordinate space computed based on ICP in the clustering step (see Fig. 1A for an example). The function *plot_pie(MigrObj)* produces a pie chart of the spot proportions in different clusters (see Fig. 1B for an example).

For comparing the different features across the clusters, their median values in each cluster can be visualized as heatmaps using the function *plot_heatmap(MigrObj)* (see Fig. 1C for an example). Function *plot_violin(MigrObj, feature = “feature name”)* can be used to inspect the distribution of a selected feature across the clusters (see Fig. 1D for an example).

The outline coordinates of cells are stored as roi_points in MigrDat object and can be used to study the typical shapes of cells in each cluster or to detect erroneously segmented cells in the experiment. Function *plot_roi(MigrObj)* plots the shapes of the selected cells as segmentation outlines allowing visual inspection of the cell shapes (see Fig. 1E for an example).

Finally, the function *plot_rag(MigrObj)* provides an opportunity to study the cell dynamics over time by plotting the cluster membership sequences or individual features on linearly arranged cell tracks (see Fig. 1F for an example). These linearized tracks allow for easy inspection of the stability of the cluster assignments or features across tracks. The default function *plot(MigrObj)* shows an overview of all spots over time connected as physical cell migration tracks, allowing for colouring of the spots based on the cluster assignment if available (see Fig. 1G for an example).

The R package CellRomeR is open source and freely available from GitHub (https://github.com/elolab/CellRomeR) together with detailed instructions and a vignette.

## 3 Results

### 3.1 Analysis of migrating HeLa cells

To illustrate the functionality of CellRomeR, we applied it to a publicly available dataset of randomly migrating HeLa cells (Holme *et al.* 2023). The majority of the imaged HeLa cells were surface attached non-polarized cells with extended protrusions, whose migratory behaviour resembles explorative movement without persistent directionality. The cells were imaged every 10 min in Incucyte S3 microscope for 14 h. The dataset was downloaded from GitHub (https://github.com/borge-holme/celltraxx_download) and the cell migration tracks were constructed with TrackMate standard Cellpose Lap Tracker workflow. In total, the data included 18 134 spots in 252 tracks after filtering in TrackMate for non-splitting tracks, with a minimum of 30 spots and a maximum of two gaps.

**Figure 2.**
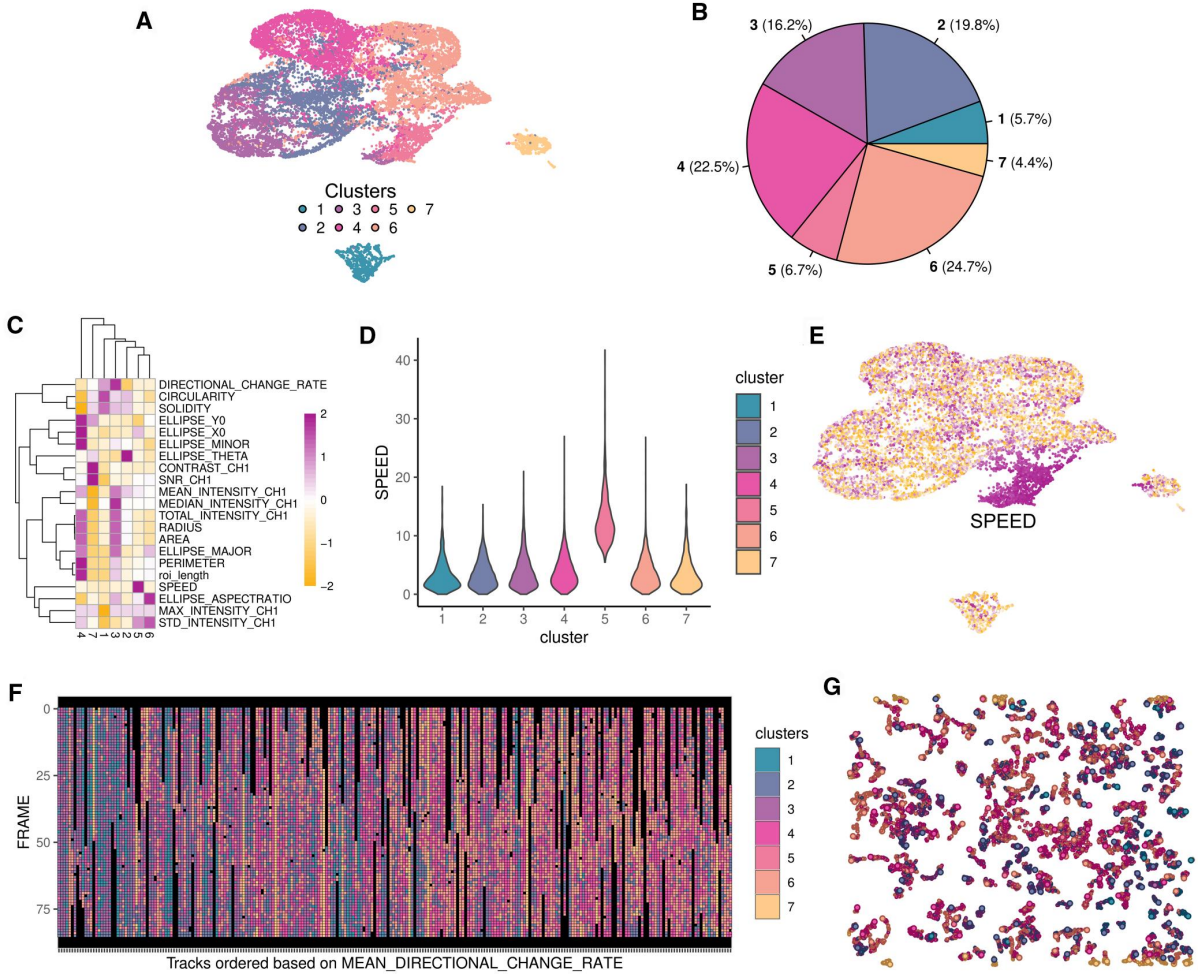
CellRomeR clustering of HeLa cells. (A) 2D representation of the clusters of migrating HeLa cells projected onto UMAP. (B) Pie chart showing the proportion of spots in each cluster. (C) Heatmap of the feature medians (rows) across the different clusters (columns). (D) Violin plots illustrating the distribution of a selected feature (speed) across the clusters. (E) Mapping of the selected feature (speed) on the 2D representation of the clustering. (F) Dynamics of the cellular phenotypes over time, with each column corresponding to one cell trajectory and the trajectories ordered based on mean directional change. (G) Overview of all spots over time, connected as physical cell migration tracks.

Using CellRomeR, we identified seven clusters of spots based on available morphological and motility features at each time point (Fig. 2A). The largest proportion (25%) of the spots belonged to cluster 6 (Fig. 2B), which showed clearest association with ellipse aspect ratio and represented typical attached HeLa cell phenotype (Fig. 2C). Summarization of the phenotypic characteristics of the clusters as a heatmap revealed heterogeneity between the different clusters (Fig. 2C). For example, speed was significantly higher in cluster 5 compared to others (Wilcoxon test *P* < .001, Fig. 2D), as also illustrated by mapping the feature to the UMAP plot (Fig. 2E). Despite the relative homogeneity of phenotypes presented in the randomly migrating HeLa cells, the clustering captured cell specific subtle but consistent differences present in the feature matrix.

Finally, dynamic analysis of the cell states over the tracks revealed both stable and variable cell trajectories, where the cell remained in the same cluster throughout the track or switched between different clusters over time, respectively (Fig. 2F). Interestingly, a large proportion (44%) of the cells showed a stable pattern (i.e. >50% of the spots in a single track belonged to a single cluster). To further illustrate the different cell states across tracks, we visualized an overview of all spots as physical cell migration tracks, coloured based on the cluster assignment (Fig. 2G).

### 3.2 Analysis of macrophages

In the second cell migration example, we applied CellRomeR clustering analysis to macrophages randomly migrating on 10 μg/ml laminin and treated with 30 μM blebbistatin to induce movement (Stinson *et al.* 2024). Blebbistatin treated macrophages exhibit morphological phenotypes varying from well attached, extended spread phenotypes to rounded poorly attached cells. The migratory behaviour varies from non-moving to explorative and to persistently migrating cells, making them suitable cells for testing clustering-based phenotypic characterization. The cells were imaged every 10 min with IX83 Olympus microscope system for 16 h. The dataset was downloaded from the supplementary material of the original study (Stinson *et al.* 2024). The cell migration tracks were constructed with TrackMate (v7.11.1) using a standard Cellpose (3.1.0) Lap Tracker workflow. TrackMate workflow was used to process image sequences to extract cell coordinates, morphological features, and cell-links in sequential images to provide cell movement tracks and feature matrices for downstream analyses. In total, the data included 3875 spots in 81 tracks after filtering in TrackMate for non-splitting tracks, with a minimum of 16 spots and a maximum of one gap.

**Figure 3.**
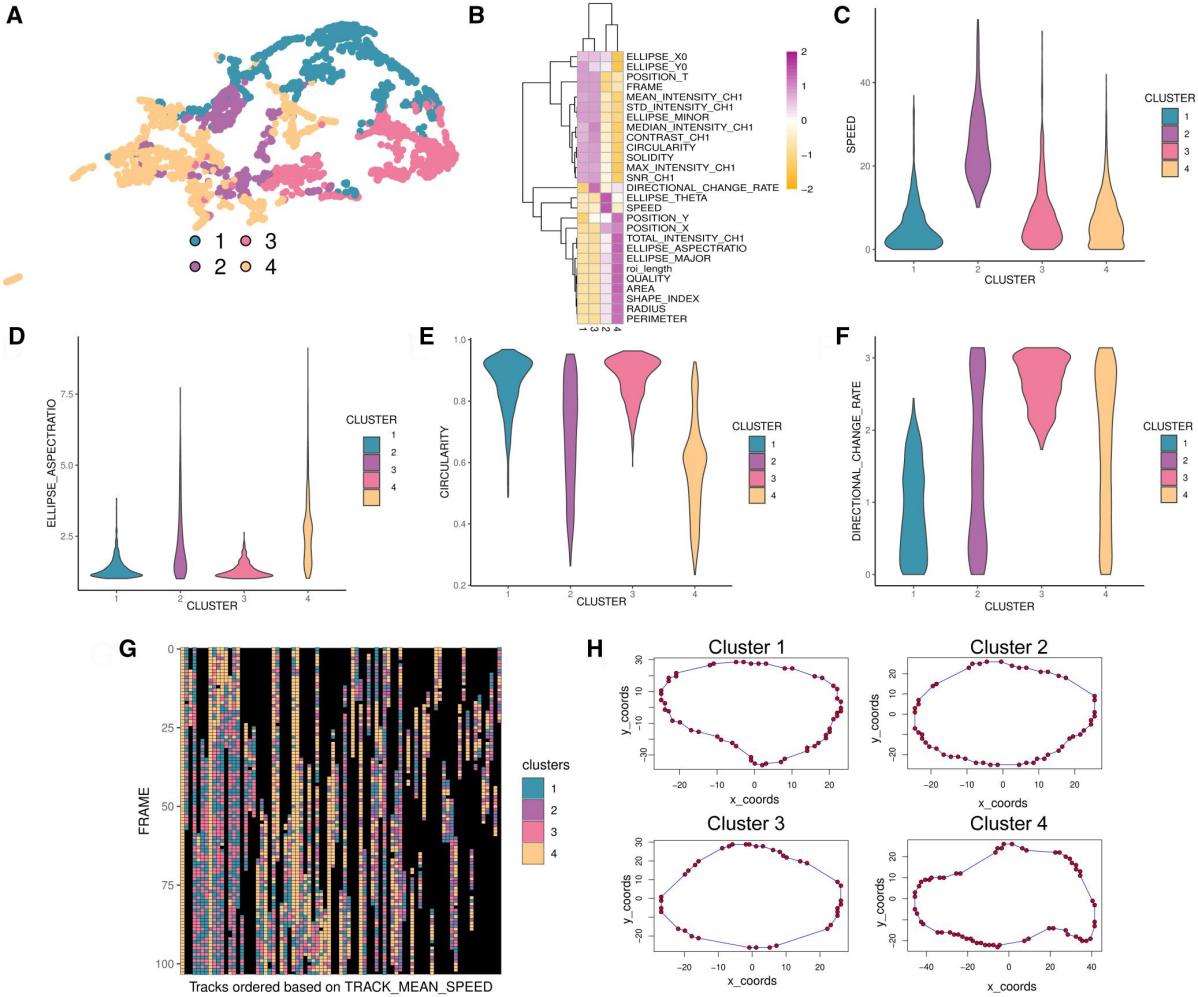
CellRomeR clustering of macrophages. (A) 2D representation of the clusters of migrating macrophages projected onto UMAP. (B) Heatmap of the feature medians (rows) across the different clusters (columns). Violin plots illustrating the distribution of (C) speed, (D) ellipse aspect ratio, (E) circularity, and (F) directionality change rate across the clusters. (G) Dynamics of the cellular phenotypes over time, with each column corresponding to one cell trajectory and the trajectories ordered based on mean directional change. (H) Representative cell shapes for each cluster.

CellRomeR clustered the macrophages into four clusters using morphological and directionality variables (Fig. 3A). These four clusters showed high concurrence with the potential biological phenotypes as inferred from the morphological and movement variables (Fig. 3B).

Similar to HeLa cells, CellRomeR clustered the macrophages with fastest directional movement into a single group, the cluster 2 (Fig. 3B and C). Macrophages in cluster 4 exhibited morphologically similar characteristics compared to cluster 2, including large area, long radius, and high ellipse aspect ratio, suggesting elongated strongly attached spreading cell phenotype (Fig. 3B and D). This suggests that while these cells were not moving fast directionally, they were still exploring their environment, as suggested by the mixed directional change rate and the morphology. Clusters 1 and 3 shared similarity of multiple morphological features including high circularity (Fig. 3B and E). However, they differed clearly on directionality change rate, in which the cells in cluster 3 showed uniformly high value (Fig. 3F). As we did not have the ground truth classifications nor did we see obvious phenotypic differences between clusters 1 and 3 in features other than directionality, we can only speculate that cluster 3 cells were more loosely attached round cells than cluster 1 cells. In conclusion, the CellRomeR grouped the macrophages into explorative, directional migration, and loosely attached round cells with intermittent surface attachment.

Like the HeLa cells, the clustering was stable along the cell tracks, as most of the macrophages in migration tracks exhibited only two phenotypes, mostly flipping between cluster 1 and 3 or cluster 2 and 4 identities (Fig. 3G). The visual inspection of cell segmentation outlines sampled for each cluster confirmed that the cells in clusters 2 and 4 showed more complex cell shapes than the cells in clusters 1 and 3 (Fig. 3H).

### 3.3 Comparison of migration analysis tools

To analyse the increasingly large and complex datasets generated by live cell imaging technologies, several tools have been developed for segmentation and tracking of cells from a series of microscopy images, such as TrackMate (Tinevez *et al.* 2017) and CellTraxx (Holme *et al.* 2023). These tools extract morphological and migratory variables from the raw microscopy data, enabling downstream analysis of cell migration patterns. However, they do not directly support analysis of the heterogeneity of cell populations or clustering of migration behaviours.

Therefore, tools have recently been developed for the analysis of numeric cell migration data downstream of TrackMate workflows, as summarized in Table 1. Among these, in addition to CellRomeR, only TrackMateR (Royle 2024) directly imports TrackMate xml data. However, it is limited to generating reports for single experiments and lacks functionality to analyse cell heterogeneity. While MigraR (Shaji *et al.* 2022) and celltrackR (Wortel *et al.* 2021) can process reformatted TrackMate exported tables, their primary focus also remains on track analysis rather than assessing cell heterogeneity along tracks or clustering cells based on spot features.

**Table 1.** Comparison of cell migration data analysis tools.

Package	Platform	Migration object	Clustering	Import TrackMate	Clustering method	Dimensional reduction	Visualization	Plots	Reference
CellRomeR	R	R S3	Cells	Yes	ICP	ICP-UMAP	Spots/tracks	UMAP, violin, heatmap, pie, tracks, and ragplot	This article
TrackMateR	R	No	No	Yes	None	None	Tracks	Tracks	Royle (2024)
celltrackR	R	No	Tracks	No	Hierarchical	PCA	Tracks	Tracks, dots	Wortel *et al.* (2021)
MigraR	R, Shiny	No	No	No	None	None	Tracks	Tracks, boxplot	Shaji *et al.* (2022)
CellPhe	R	No	Cells	No	Hierarchical, k-means	PCA	Spots/tracks	Multiple	Wiggins *et al.* (2023)
cellPLATO	Python	No	Cells/tracks	No	HDBSCAN on UMAP	UMAP	Spots/tracks	Multiple	Shannon *et al.* (2024)
Traject3D	KNIME (R, Python)	No	Cells	No	PhenoGraph	None	Spots/tracks	Multiple	Freckmann *et al.* (2022)
TrackMate	ImageJ, MATLAB	XML	No	No	None	No	Tracks	Multiple	Tinevez *et al.* (2017)
CellTraxx	ImageJ, C	No	No	No	None	No	Tracks	Tracks, dots	Holme *et al.* (2023)
CellMissy	Java	Database	No	No	None	None	None	None	Masuzzo *et al.* (2017)

Only recently, tools such as Traject3D (Freckmann *et al.* 2022), CellPhe (Wiggins *et al.* 2023), and cellPLATO (Shannon *et al.* 2024) have been introduced to enable clustering-based identification of subsets of similarly behaving cells, enhancing the analysis of cellular heterogeneity and migration patterns. While these tools represent important advancements, they typically rely on basic clustering techniques, which may limit their ability to capture complex migration behaviours. Additionally, their usability, user-friendliness, and range of functionalities vary. For instance tools, such as cellPLATO, offer various analytical and visualization functions but are primarily Python-based, restricting their usability in R-based workflows.

CellRomeR provides a unique cell migration analysis tool in the R environment, including a new approach to identify cell subsets, building on an iterative clustering projection method, as well as multiple visualization options to enable in-depth study of cellular behaviour. Additionally, it introduces a dedicated migration data object for migration data and analysis results. The open source migration data object allows its flexible use also in future applications with other types of cell microscopy data.

## 4 Conclusions

In conclusion, CellRomeR offers a comprehensive workflow that integrates the visualization and quantification of cell tracks with the analysis of cell behaviours based on advanced clustering based on self-supervised machine learning. This enables in-depth interpretation of cellular dynamics and heterogeneity to enhance our understanding of complex biological processes.

## Data Availability

The cell migration datasets used in this article were downloaded from GitHub (https://github.com/borge-holme/celltraxx_download) and webpage of the original study (Stinton *et al.* 2024) at molbiolcell.org (https://doi.org/10.1091/mbc.E23-04-0137).
